# 336. Microbiological Outcomes of Oral Omadacycline Treatment in Adults with Nontuberculous Mycobacterial Pulmonary Disease (NTM-PD) Caused by Mycobacterium abscessus complex (MABc): Results from a Phase 2, Double-blind, Randomized, Placebo-controlled, Multi-center Trial

**DOI:** 10.1093/ofid/ofaf695.119

**Published:** 2026-01-11

**Authors:** Reeti Khare, Diane M Anastasiou, Surya Chitra, Alisa W Serio

**Affiliations:** National Jewish Health, Denver, CO; Paratek Pharmaceuticals, Inc., King of Prussia, PA; Paratek Pharmaceuticals, Inc., King of Prussia, PA; Paratek Pharmaceuticals, Inc., King of Prussia, PA

## Abstract

**Background:**

Nontuberculous mycobacterial pulmonary disease (NTM-PD) is a chronic disease commonly caused by *Mycobacterium abscessus* (MAB; previously *M. abscessus* complex; which is comprised of 3 subspecies: *abscessus*, *massiliense*, *bolletii*). Treatment of NTM-PD due to MAB is challenging due to intrinsic drug resistance, lack of effective oral antibiotics, and a need for complex, lengthy therapies. We describe microbiological endpoints from a double-blind, placebo-controlled clinical trial of oral omadacycline in NTM-PD due to MAB (NCT04922554).

**Figure 1.**
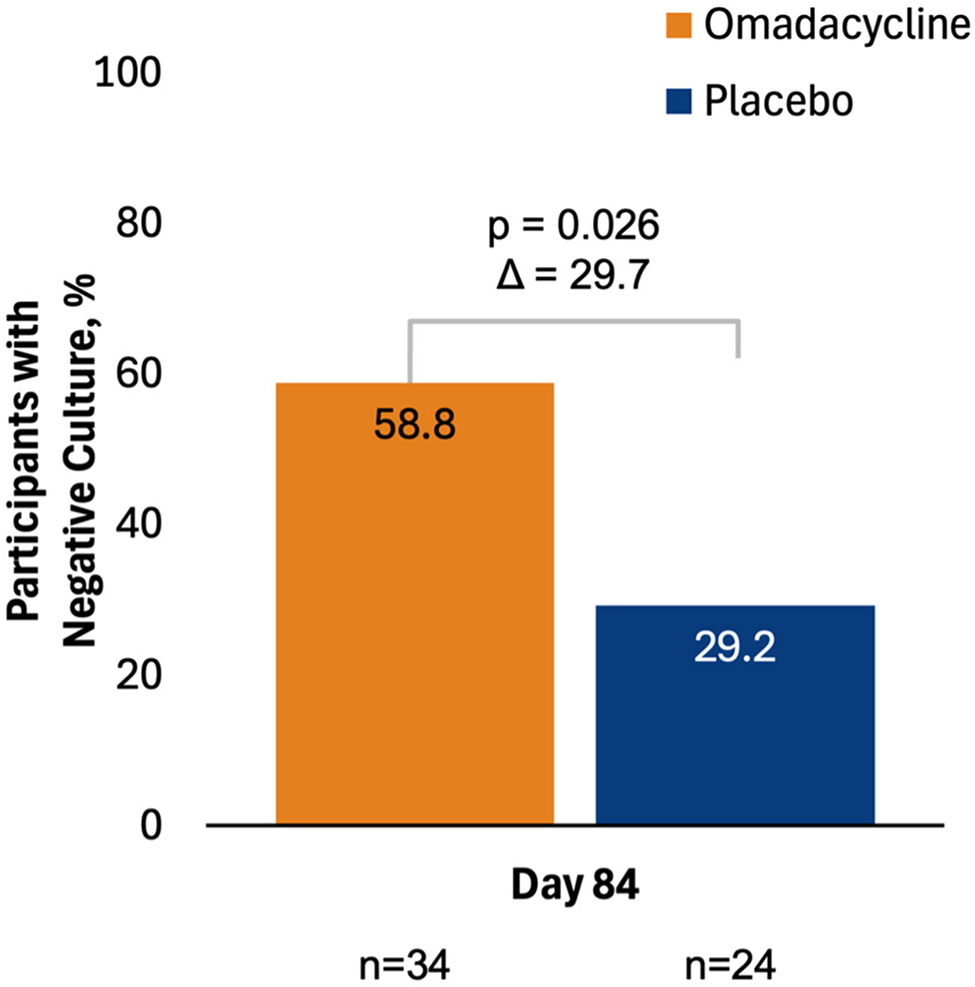
Negative M. abscessus sputum cultures (both broth and agar) at Day 84 (exploratory endpoint).

**Figure 2.**
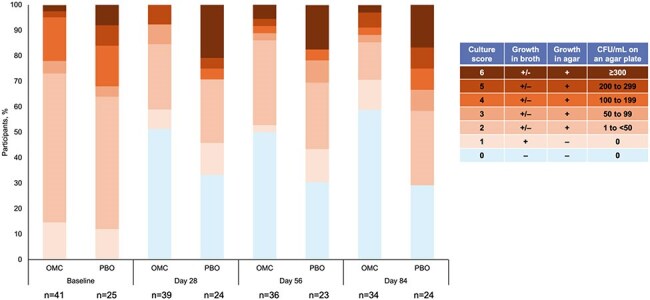
Semi-quantitative sputum culture scores of M. abscessus in liquid media and on agar plates at baseline and post-baseline assessment points (exploratory endpoint). Scoring table adapted from: Griffith DE, et al. Am J Respir Crit Care Med. 2015 Sep 15;192(6):754-60.

**Methods:**

Adults with NTM-PD caused by MAB were randomized 1.5:1 to receive 300 mg oral omadacycline monotherapy once daily (OMC, n=41) or placebo (PBO, n=25) for 84 days. Eligible patients met diagnostic criteria for MAB NTM-PD with no need for guideline-directed antibiotic therapy within 3 months and were excluded if they had antibiotic treatment for NTM in the prior 6 months, any systemic/inhaled antibiotic therapy (except chronic macrolides) within prior 4 weeks, cystic fibrosis, cavitary disease, or extrapulmonary NTM disease. Microbiological endpoints assessed negative sputum cultures (Day 84) and bacterial burden.

**Figure 3.**
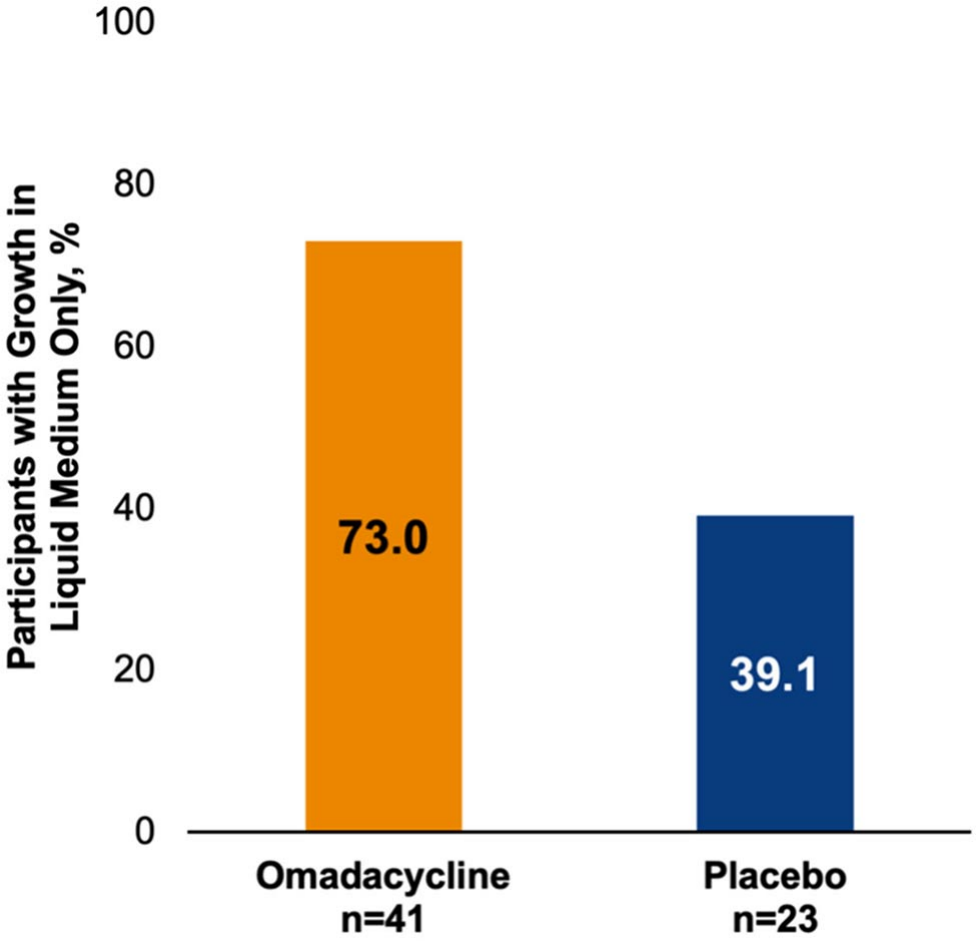
Participants with M. abscessus growth in liquid medium only (SSC score=1) and no growth in agar at any timepoint (secondary endpoint).

**Figure 4.**
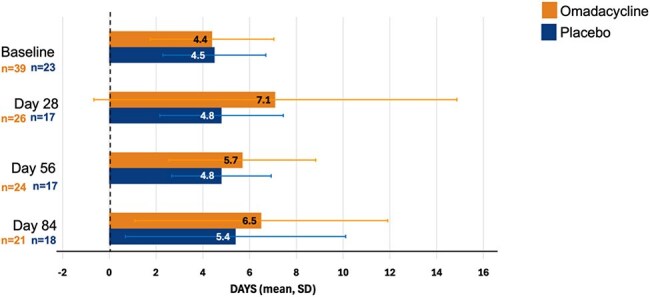
Mean time (days) to detection of M. abscessus growth in liquid media (exploratory endpoint). Error bars show 1 standard deviation.

**Results:**

At Day 84, 58.8% of OMC-treated participants versus 29.2% of PBO-treated participants had negative sputum cultures (Figure 1). Semi-quantitative sputum culture (SSC) scores at baseline were similar for both cohorts; post-baseline at every timepoint, there was a greater decrease in score for OMC-treated participants versus PBO-treated participants (Figure 2). Overall, more participants in the OMC group had cultures that grew in liquid medium only with no growth in agar (SSC score=1) vs PBO at any timepoint (Figure 3). At baseline, mean time to detection (in days) of MAB growth in liquid media was similar in both cohorts (OMC, 4.4; PBO, 4.5) but at all other timepoints was longer in the OMC cohort versus PBO, respectively (Day 28, 7.1 vs. 4.8; Day 56, 5.7 vs. 4.8; Day 84, 6.5 vs. 5.4; Figure 4).

**Conclusion:**

Treatment with OMC resulted in more negative cultures at Day 84 than PBO. Data suggest that OMC treatment decreased the mycobacterial burden of infection in patients with NTM-PD caused by MAB by a greater reduction in SSC scores, more participants with an SSC score=1 versus PBO, and longer time to positive cultures in liquid media.

**Disclosures:**

All Authors: No reported disclosures

